# Objective Skin Quality Assessment after Reconstructive Procedures for Facial Skin Defects

**DOI:** 10.3390/jcm11154471

**Published:** 2022-07-31

**Authors:** Dinko Martinovic, Slaven Lupi-Ferandin, Daria Tokic, Mislav Usljebrka, Andrija Rados, Ante Pojatina, Sanja Kadic, Ema Puizina, Ante Mihovilovic, Marko Kumric, Marino Vilovic, Dario Leskur, Josko Bozic

**Affiliations:** 1 Department of Maxillofacial Surgery, University Hospital of Split, 21000 Split, Croatia; dmartinovic@kbsplit.hr (D.M.); slupi@mefst.hr (S.L.-F.); musljebrka@kbsplit.hr (M.U.); arados@kbsplit.hr (A.R.); apojatina@kbsplit.hr (A.P.); skadic@kbsplit.hr (S.K.); epuizina@kbsplit.hr (E.P.); amihovilovic@kbsplit.hr (A.M.); 2 Department of Pathophysiology, University of Split School of Medicine, 21000 Split, Croatia; marko.kumric@mefst.hr (M.K.); marino.vilovic@mefst.hr (M.V.); 3 Department of Anesthesiology and Intensive Care, University Hospital of Split, 21000 Split, Croatia; dtokic@kbsplit.hr; 4 Department of Pharmacy, University of Split Schwool of Medicine, 21000 Split, Croatia; dario.leskur@mefst.hr

**Keywords:** skin flap, skin graft, skin quality, reconstructive surgery, facial surgery

## Abstract

Local random skin flaps and skin grafts are everyday surgical techniques used to reconstruct skin defects. Although their clinical advantages and disadvantages are well known, there are still uncertainties with respect to their long-term results. Hence, the aim of this study was to evaluate outcomes more than one-year post operatively using objective measurement devices. The study included 31 facial defects reconstructed with local random flap, 30 facial defects reconstructed with split-thickness skin grafts (STSGs) and 30 facial defects reconstructed with full-thickness skin grafts (FTSGs). Skin quality was objectively evaluated using MP6 noninvasive probes (Courage + Khazaka GmbH, Cologne, Germany), which measure melanin count, erythema, hydration, sebum, friction and transepidermal water loss. The results showed that there were no significant differences in melanin count, erythema, hydration, sebum level, friction value and transepidermal water loss (TEWL) between the site reconstructed with random local flaps and the same site on the healthy contralateral side of the face. However, both FTSGs and STSGs showed significantly higher levels in terms of TEWL and erythema, whereas the levels of hydration, sebum and friction were significantly lower compared to the healthy contralateral side. Moreover, STSGs resulted in a significant difference in melanin count. These findings imply that the complex pathophysiology of the wound-healing process possibly results in better skin-quality outcomes for random local flaps than skin autografts. Consequently, this suggests that random local flaps should be implemented whenever possible for the reconstruction of facial region defects.

## 1. Introduction

Reconstruction of skin defects is one of the oldest surgical techniques most commonly performed after traumatic injuries or oncological excisions. Apart from primary closure and secondary healing, there is a broad range of possible reconstruction methods, such as skin autografts, local flaps, distant/regional flaps, and microvascular free tissue transfer [1,2,3]. The facial region is especially sensitive with respect to the selection of an appropriate reconstruction method to provide functional and aesthetically pleasing results. Whereas larger and profounder defects that affect several types of tissues are usually reconstructed using distant and regional flaps or more modern techniques, such as free flaps, somewhat smaller and superficial defects can be reconstructed using random local flaps or skin autografts [4,5].

Skin autografts are autotransplants from a patient’s donor site to the defect site. Depending on the thickness, they can be divided into split-thickness skin grafts (STSGs) and full-thickness skin grafts (FTSGs) [6,7]. STSGs involve the epidermis and part of the underlying dermis, whereas FTSGs involve the epidermis and the entire dermis [8]. FTSG is usually chosen for small defects when the best possible aesthetic result is needed; in the facial region, FTSG is usually used for the nose, ear or eyelid. On the other hand, STSG is usually used to covering somewhat diametrically larger defects in the temporal region, forehead or the scalp. However, depending on the patient’s status and the defect, both FTSGs and STSGs can be used in various regions [9]. Skin autografts do not have an initially autonomous blood supply, and in the first 48–72 h, they are bound to the absorbing transudate from the recipient site, a process called plasmatic imbibition [10]. Consequently, they can only survive on tissues such as the subcutis, periosteum, perichondrium and muscles, which can provide them with nutrients through the transudate. During the first days after grafting, the capillary buds start the revascularization phase, which should be completed within 5–7 d [11]. Then, the remodeling phase starts, wherein the graft undergoes retraction, adjustment and reinnervation [12].

Local random flaps are full-thickness skin with the subcutaneous layer sectioned and detached on all except one side (usually one lateral side); however, in certain types of flaps, the base serves as the only attachment (called the peduncle), and the flap vitality is determined by its vascularization [13,14,15,16]. After reconstruction, flaps adapt to the reduced vascularization, but over time, the blood supply increases due to the hyperplasia of the peduncle circulation and neovascularization from the wound margins [17]. Whereas arterial flaps survive with the help of a specific artery, random flaps depend on random circulation through the superficial subcutaneous layer, which is the richest in the facial region.

Both skin autografts and local random flaps are among the most commonly used reconstruction techniques in plastic surgery of the facial region. However, there are still some uncertainties with respect to their healing processes, especially regarding their long-term results. Most relevant data regarding the advantages and disadvantages of these methods were established in the 20th century [18]. According to the principle of reconstruction of “the same from the same”, local random flaps should be more aesthetically pleasant than skin autografts for the facial region [19]. Furthermore, discoloration of the recipient site is a considered a major weakness of skin autografts compared local random flaps [20]. Moreover, local random flaps usually produce a lesser degree of scaring due to the absence of a strong secondary contraction, which is prominent in skin autografts, especially STSGs. Furthermore, skin flaps and FTSGs involve all skin appendages, whereas STSGs do not. However, none of these established advantages and disadvantages have been evaluated directly on the skin using an objective instrument after the wound-healing remodeling phase (>year after the procedure).

Hence, the primary aim of this study was to evaluate objective skin quality parameters in the facial region following reconstruction with local random flaps, FTSGs and STSGs. The secondary goal was to evaluate these same objective parameters on the healthy contralateral side of the face for comparison with the reconstructed area and to compare their differences (Δ) between the aforementioned reconstruction methods.

## 2. Materials and Methods

### 2.1. Study Design and Ethical Considerations

This cross-sectional study was performed at the Department of Maxillofacial Surgery, University Hospital of Split, during the time period from June 2021 to January 2022.

All subjects were informed about the purpose and procedures of the study in a timely manner, and they all signed an informed consent to participate. The study was approved by the Ethics Committee of the University Hospital of Split and conducted in accordance with the latest version of the Declaration of Helsinki.

### 2.2. Subjects

The study included 31 facial defects reconstructed with local random flap, 30 facial defects reconstructed with STSGs and 30 facial defects reconstructed with FTSGs. Participants were recruited to the study during control check-ups. All included participants underwent an operation due to basal cell carcinoma (BCC) or squamous cell carcinoma (SCC). Furthermore, all three reconstructive procedures were conducted at the Department of Maxillofacial Surgery, University Hospital of Split, according to the standard surgical protocols and guidelines. Our institution prefers local skin flaps over skin autografts for facial reconstruction. Indications for use of FTSGs were the nasal and eyelid areas, whereas the indication for use of STSGs was diametrically larger defects (>40 mm) in the frontotemporal and forehead areas. However, these indications are individually dependent on the patient’s age, status and skin elasticity.

The inclusion criteria for participants were age of 18–90 years, >1 year since reconstruction, no postoperative complications and a healthy contralateral side of the face. According to most authors, skin healing and remodeling is completed one year after reconstruction. Additionally, only patients with FTSGs from supraclavicular donor sites were included, as well as only STSGs from upper-arm donor sites (0.4 mm thickness during harvest).

Exclusion criteria were paramedial location of the reconstructed defect, recidivism of the malignancy, re-excision of the reconstructed site, other active malignant diseases, diabetes mellitus, chronic dermatological diseases, smoking, excessive alcohol consumption and psychiatric diseases. Prior to inclusion, all subjects underwent a detailed physical examination and meticulous inspection of their anamnestic data.

### 2.3. Objective Skin Assessment

Skin quality was objectively evaluated by the same experienced investigator using an MP6 skin quality assessment instrument (Courage + Khazaka GmbH, Cologne, Germany). The instrument assesses several skin qualities using noninvasive probes. Transepidermal water loss (TEWL) was assessed as an objective sign of skin barrier function using a Tewameter^®^ TM 300 instrument. Skin hydration was assessed using a Corneometer^®^ CM 825. The amount of erythema and melanin was measured using a Mexameter^®^ MX 18. Skin friction was estimated using a Frictiometer^®^ FR 700. Sebum was measured using a Sebumeter^®^ SM 815. All probes were calibrated according to the manufacturers’ instructions before study onset.

All participants underwent measurements in a room with stable conditions. Air humidity was kept at 40–55% using a Philips 3000i air humidifier (Koninklijke Philips N.V., Amsterdam, the Netherlands), and the room temperature was kept at 20–22 °C using the inbuilt hospital air conditioners. The participants were first seated in the room for 20 min to acclimate the skin to the aforementioned conditions. Participants were instructed to take a shower the morning of the measurement day and to strictly avoid using any make-up, skin creams or any other skin preparations. Probes were used to measure both the reconstructed area and the same area on the contralateral healthy side. The probes were held at a right angle and gently applied to the skin for optimal contact, and all measurements were performed three times, after which the mean value was calculated. After every participant, probes were disinfected and prepared for the next subject.

The same site on the healthy contralateral side was measured as a referent value, and to diminish the interparticipant variability, we computed the difference (Δ) between the healthy and reconstructed site (Δ = healthy site parameter—reconstructed site parameter).

Moreover, to test for possible intraobserver variability, several participants underwent objective evaluation with the probes on three (3) different days. There was no statistically significant difference between these results.

### 2.4. Statistical Analyses and Sample Size Calculation

All data analyses were performed using MedCalc statistical software (MedCalc Software, version 20.110, Ostend, Belgium). Qualitative variables are presented as whole numbers and percentages. Continuous quantitative data are presented as mean ± standard deviation, whereas non-continuous data are presented as median and interquartile range. The normality of the data distribution was estimated using the Kolmogorov–Smirnov test. A chi-square test was used for comparison of categorical variables. A student’s *t*-test was used for comparison of parametric variables, whereas a Mann–Whitney U test was used for comparison of non-parametric variables. One-way analysis of variance (ANOVA) with post hoc Tukey’s test was used for comparison of parametric variables between groups, whereas Kruskal–Wallis test with post hoc Dunn’s test was used for comparison of non-parametric variables between groups. The level of statistical significance was set *p* < 0.05.

Sample size was analyzed using the data from a pilot study on 15 randomly selected subjects from the patient population (5 patients with random flaps, 5 patients with FTSGs and 5 patients with STSGs). Melanin count difference (Δ), which was one the main outcomes of the study, was used for the calculation. In random flap patients, the mean melanin Δ was 2.0 ± 6.0 AU, whereas in FTSG patients, it was −6.1 ± 7.0 AU, and in STSG patients, it was −29.0 ± 15 AU. With a type I error of 0.05 and a power of 90%, the required sample size was 15 participants per group.

## 3. Results

There were 50 (55.0%) male and 41 (45.0%) female participants included, and the mean age of the study population was 78.6 ± 8.3 years. With respect to the defect diameter, STSGs had the largest size, with a significant difference relative to the other two groups (*p* < 0.001). There were no other significant differences between the three groups with respect to the anthropometric and clinical characteristics (Table 1).

There were no significant differences with respect to the objective skin parameters between the random local flaps and the healthy contralateral side for all tested parameters (Table 2). However, both FTSG and STSG and patients presented a significantly higher erythema level and TEWL, and both groups showed a significantly lower level of hydration, sebum and friction compared to the healthy contralateral side (Table 2). However, only STSG patients presented significantly higher melanin counts (*p* < 0.001) (Table 2).

**Figure 1:**
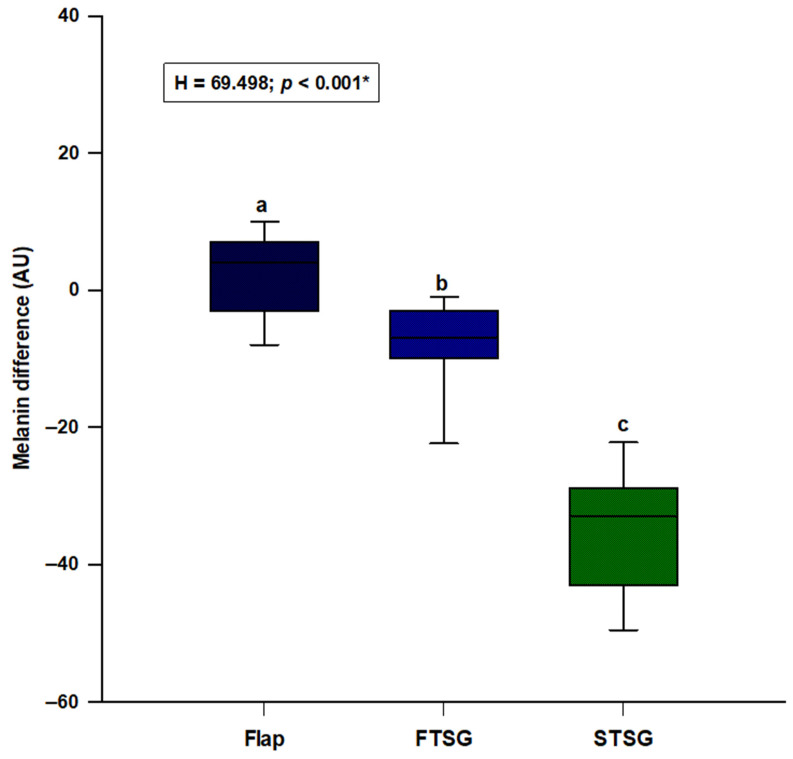
Comparison of the melanin difference between the random local flaps (N = 31), STSG (N = 30) and FTSG (N = 30) groups. **Abbreviations: FTSG**—full-thickness skin graft; **STSG**—split-thickness skin graft. * Kruskal–Wallis test with post hoc Dunn’s test. a vs. b—*p* < 0.05; a vs. c—*p* < 0.05; b vs. c—*p* < 0.05.

After calculating the difference (Δ) of the skin parameters between the healthy and the reconstructed site, we compared them between the local random flaps, FTSG and STSG groups. We observed a significant difference in melanin (H = 69.498; *p* < 0.001), with random skin flaps resulting in the lowest difference and STSG with the highest difference (flaps: 4.0 (−3.0–7.0); FTSG: −7.0 (−10.0–−3.0); STSG: −33.0 (−43.0–−29.0)) (Figure 1). Post hoc analysis showed a significant difference between all three groups (*p* < 0.05) (Figure 1).

**Figure 2:**
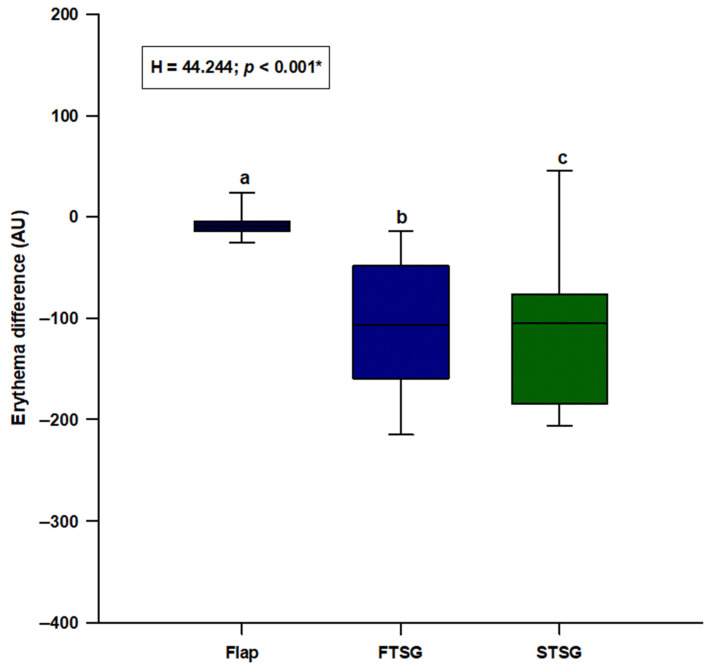
Comparison of the erythema difference between the random local flaps (N = 31), STSG (N = 30) and FTSG (N = 30) groups. **Abbreviations: FTSG**—full-thickness skin graft; **STSG**—split-thickness skin graft. * Kruskal–Wallis test with post hoc Dunn’s test. a vs. b—*p <* 0.05; a vs. c—*p <* 0.05; b vs. c—*p >* 0.05.

We observed a statistically significant difference in erythema levels (H = 44.244; *p* < 0.001), with the random skin flap group showing the lowest difference and the STSG group presenting the highest difference (flaps: −9.0 (−14.0–−4.0); FTSG: −107.0 (−160.0–−48.0); STSG: −105.0 (−184.0–−76.0)) (Figure 2). Post hoc analysis showed a significant difference between random skin flaps and both FTSG (*p* < 0.05) and STSG (*p* < 0.05) (Figure 2).

**Figure 3:**
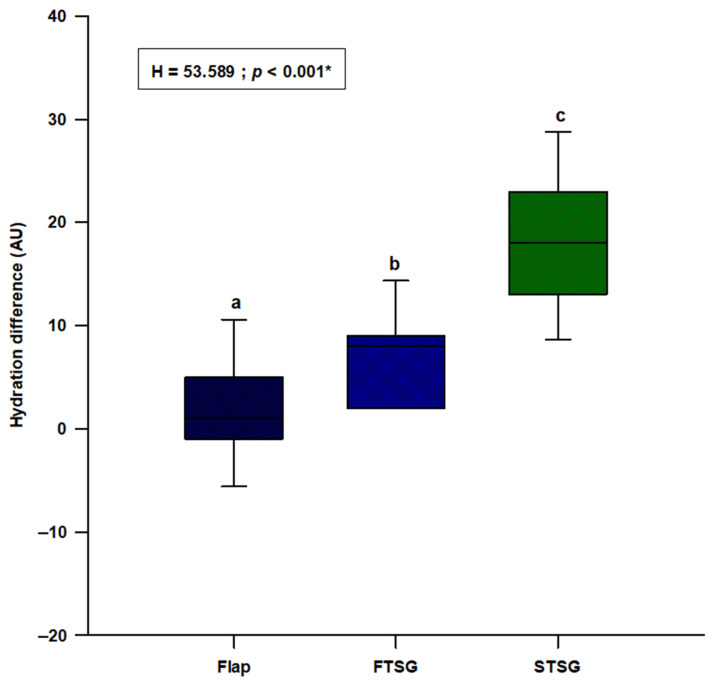
Comparison of the hydration difference between the random local flaps (N = 31), STSG (N = 30) and FTSG (N = 30) groups. **Abbreviations: FTSG**—full–thickness skin graft; **STSG**—split-thickness skin graft. * Kruskal–Wallis test with post hoc Dunn’s test. a vs. b—*p <* 0.05; a vs. c—*p <* 0.05; b vs. c—*p <* 0.05.

We observed a statistically significant difference in hydration (H = 53.589; *p* < 0.001), with the random skin flap group showing the lowest difference and the STSG group presenting the highest difference (flaps: 1.0 (−1.0–5.0); FTSGs: 8.0 (2.0–9.0); STSGs: 18.0 (13.0–23.0)) (Figure 3). Post hoc analysis showed a significant difference between all three groups (*p* < 0.05) (Figure 3).

**Figure 4:**
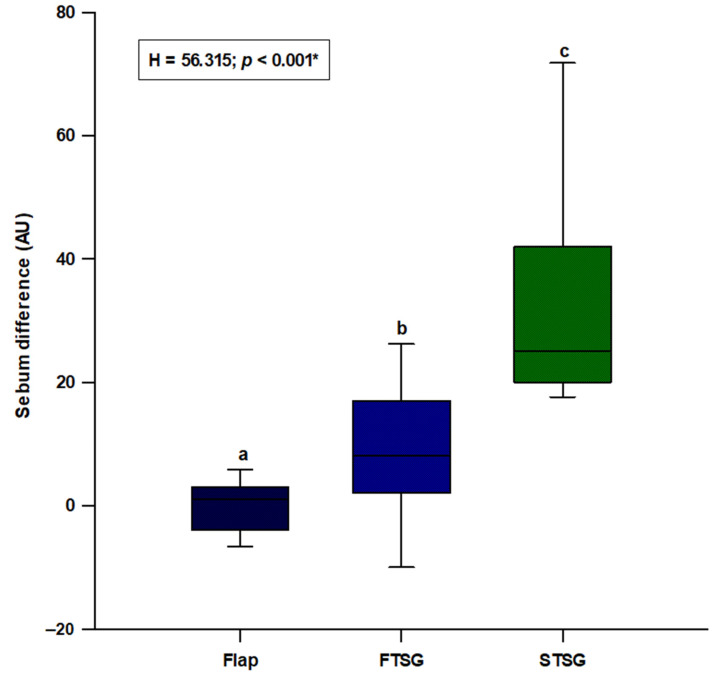
Comparison of the sebum difference between the random local flaps (N = 31), STSG (N = 30) and FTSG (N = 30) groups. **Abbreviation: FTSG**—full–thickness skin graft; **STSG**—split-thickness skin graft. * Kruskal–Wallis test with post hoc Dunn’s test. a vs. b—*p <* 0.05; a vs. c—*p <* 0.05; b vs. c—*p <* 0.05.

We observed a statistically significant difference in sebum (H = 56.315; *p* < 0.001), with the random skin flap group showing the lowest difference and the STSG group presenting the highest difference (flaps: 1.0 (−4.0–3.0); FTSGs: 8.0 (2.0–17.0); STSGs: 25.0 (20.0–42.0)) (Figure 4). Post hoc analysis showed a significant difference between all three groups (*p* < 0.05) (Figure 4).

**Figure 5:**
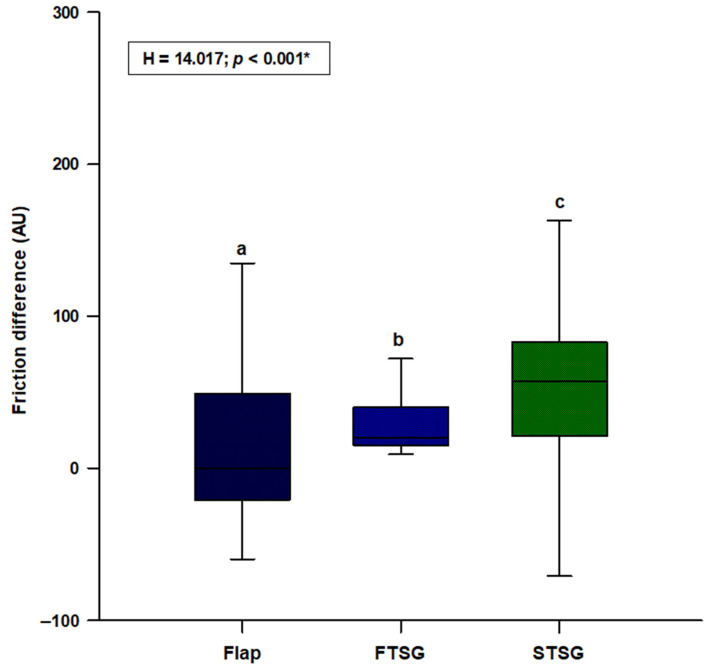
Comparison of the friction difference between the random local flaps (N = 31), STSGs (N = 30) and FTSGs (N = 30). **Abbreviation: FTSG**—full–thickness skin graft; **STSG**—split-thickness skin graft. * Kruskal–Wallis test with post hoc Dunn’s test. a vs. b—*p >* 0.05. a vs. c—*p <* 0.05. b vs. c—*p >* 0.05.

We observed a statistically significant difference in friction (H = 14.017; *p* < 0.001), with the random skin flap group showing the lowest difference and the STSG group presenting the highest difference (flaps: 0.0 (−21.0—49.0); FTSGs: 20.0 (15.0—40.0); STSGs: 51.0 (21.0—83.0)) (Figure 5). Post hoc analysis showed a significant difference between random skin flaps and STSGs (*p* < 0.05) (Figure 5).

**Figure 6:**
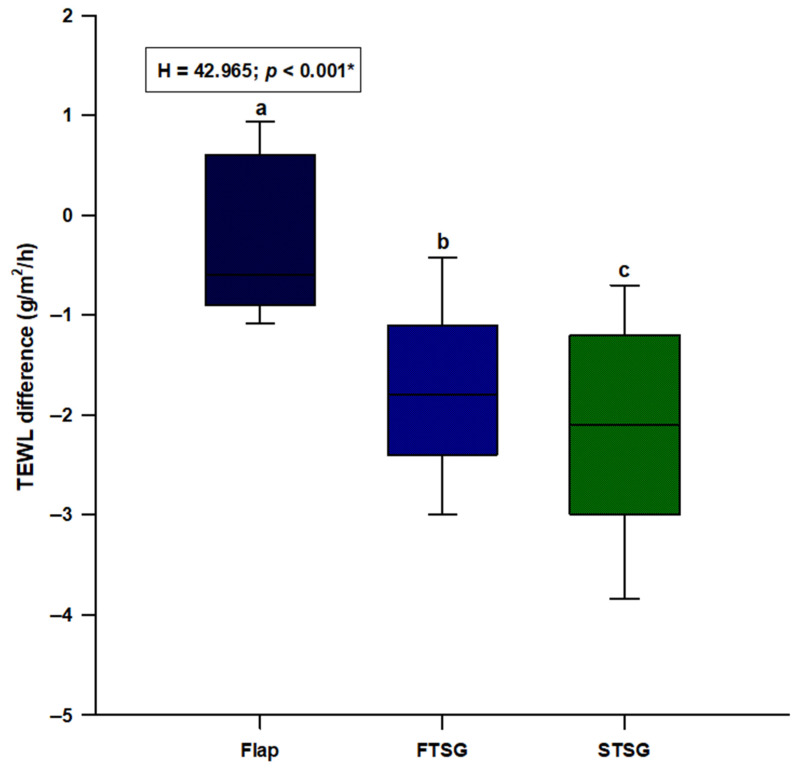
Comparison of the TEWL difference between the random local flaps (N = 31), STSG (N = 30) and FTSG (N = 30) groups. **Abbreviations: FTSG**—full–thickness skin graft; **STSG**—split-thickness skin graft; **TEWL**—transepidermal water loss. * Kruskal–Wallis test with post hoc Dunn’s test. a vs. b—*p <* 0.05; a vs. c—*p <* 0.05; b vs. c—*p >* 0.05.

TEWL showed a statistically significant difference (H = 42.965; *p* < 0.001) with random skin flaps showing the lowest difference and STSGs presenting the highest difference (Flaps: −0.6 (−0.9–0.6); FTSGs: −1.8 (−2.4–−1.1); STSGs: −2.1 (−3.0–−1.2)) (Figure 6). Post hoc analysis showed a significant difference between random skin flaps and both the FTSG (*p* < 0.05) and STSG (*p* < 0.05) groups (Figure 6).

## 4. Discussion

The results of this study showed that there were no significant differences in melanin count, erythema, hydration, sebum level, friction value and TEWL between the site reconstructed with random local flaps and the same site on the healthy contralateral side of the face. However, both the FTSG and STSG groups had significantly higher levels of TEWL and erythema, whereas hydration, sebum and friction levels were significantly lower compared to the healthy contralateral side. The STSG group also had higher melanin counts. With respect to differences (Δ) between the healthy and reconstructed site, the results showed a significant difference between the three reconstruction methods in all parameters. Moreover, post hoc analyses revealed that the random local flaps group had the lowest Δ, whereas the STSG group had the highest Δ regarding all evaluated skin quality parameters. Based on an extensive search of the available literature, we concluded that this is the first study to objectively compare skin quality between local random flaps, FTSG and STSG.

The results of our objective skin evaluations are partially in line with the recognized advantages and disadvantages of local flaps, FTSG and STSG. Discoloration is one of the main aesthetic disadvantages of FTSG and STSG for facial reconstruction [21,22]. However, FTSG causes a lesser degree of discoloration than STSG when harvested from the head and neck regions, such as the supraclavicular, retroauricular or scalp regions. Although FTSG still results in a certain degree of discoloration in comparison to healthy facial skin, our results showed that there was no statistically significant difference. Whereas some authors have mentioned hypopigmentation as a possible skin graft outcome, most agree that hyperpigmentation is the most frequently exhibited trait; however, the pathophysiology behind this phenomenon is still unclear. [23,24]. Whereas it seems that the grafted skin displays a higher melanin count compared to healthy skin, a study by Tsukada et al. showed that histologically, the melanocyte count was much lower in the skin graft group [25]. The proposed explanation for this paradox is that the contraction of the graft could lead to a closer approximation of melanin in the skin [26].

The contraction of has been skin grafts was well-established in numerous studies [27,28]. Whereas primary graft contraction occurs immediately after the harvest due to the passive recoil of the elastin fibers, secondary contraction occurs over time after the reconstruction due to myofibroblasts in the wound bed [28]. FTSG results in greater primary contraction owing it to the larger amount of dermis, whereas STSG results in greater secondary contraction owing it to the lesser amount of dermis, which consequently increases susceptibility to myofibroblast pulling [27]. As mentioned previously, contracture is the possible cause of skin graft hyperpigmentation, and it could also possibly influence the friction quality of the skin. Our results showed that the friction value is lowest in the STSG group and somewhat higher in FTSG group, whereas the friction was most similar to that of healthy skin in the local flaps group. These results are contrary to those reported in a study conducted on finger pads, which showed that even a small degree of tangential skin stretch, a trait equivalent to contraction, resulted in increased perceived friction [29]. Moreover, this could be especially interesting regarding our results indicating that skin grafts resulted in significantly lower hydration compared to healthy skin. It is well-established that skin hydration, which is provided by the stratum corneum, is one of the main factors that contribute to a higher skin friction value [30,31]. It is possible that the hydration from stratum corneum plays a greater role in skin friction than contraction similar to tangential stretch. Nevertheless, this hypothesis needs to be addressed in future studies.

As TEWL is one of the most important indicators of skin barrier function, it is an important and interesting subject with respect to skin grafts. Our results showed significantly higher TEWL in both the FTSG and STSG groups compared to the healthy side. Some of our results are contrary to those reported in a study conducted by Kim et al. wherein objective measurements were used to follow-up skin changes in STSG patients [26]. Their study showed that although there were paradoxical dynamics during months of follow-up, one year after the procedure, STSG patients did not show any statistically significant change in skin function, although TEWL and epidermal hydration levels were somewhat reduced. Another interesting point of view is presented in a study by Suetake et al., who found that keloid and hypertrophic scars have higher TEWL levels than normal skin subsequent to aberrations of the stratum corneum [32]. Although keloids and hypertrophic scars have different pathophysiological mechanisms than skin grafts, it is possible that they both exhibit functional abnormalities of the stratum corneum.

Another result of this study that should be highlighted is the lower sebum levels found in skin autografts, most prominently in STSG patients. The dermis contains connective tissue and skin appendages, such as sebaceous glands, hair follicles and sweat glands. Although sebaceous glands are seated in the dermis, hair follicles and sweat glands extend into subcutaneous fat. Studies have shown that transplanted appendages survive in skin grafts; however, because only a part of the dermis is included, STSG patients often have functionally deficient sebaceous glands, sweat glands and hair follicles [33]. Sebum also plays an important role as a skin barrier facilitator. A study conducted on radiation-induced skin injury showed that the atrophy of sebaceous glands had a considerable impact on TEWL and skin hydration [34].

There are several limitations to our study. First of all, we were not able to eliminate all of the confounding effects, and the cross-sectional design prohibited making any causal conclusions. Moreover, our sample size was relatively small, and the study was conducted in a single center. Additionally, the instrument used for objective skin quality assessments has a noted interobserver variability. We mitigated this limitation by using only one experienced investigator for all instrumental evaluations.

## 5. Conclusions

Our results showed that after the remodeling phase (>1 year postoperative), random local skin flaps resulted in significantly better skin quality than STSG and FTSG. These findings imply that the complex pathophysiology of the wound-healing process possibly results in better skin quality outcomes for random local flaps than skin autografts. Moreover, these outcomes suggest that random local flaps should be implemented in the reconstruction of the facial region defects when permitted by the reconstruction site and size of defect. However, larger multicentric, longitudinal studies are needed to further address our findings.

## Figures and Tables

**Table 1:** Anthropometric and clinical characteristics of the study sample.

Parameter	Study Population (N = 93)	Flaps (N = 31)	FTSGs (N = 30)	STSGs (N = 30)	*p*
Male gender (N,%)	51 (55.0)	17 (54.8)	18 (60.0)	16 (53.3)	0.861 *
Age (years)	78.6 ± 8.3	76.5 ± 7.0	79.3 ± 9.0	79.8 ± 8.6	0.243 ^†^
Body height (cm)	179.1 ± 10.8	178.2 ± 9.6	177.2 ± 7.5	181.0 ± 11.1	0.136 ^†^
Body mass (kg)	78.2 ± 8.9	77.6 ± 8.7	76.5 ± 10.9	80.4 ± 6.3	0.199 ^†^
BMI (kg/m^2^)	24.5 ± 2.7	24.7 ± 2.8	24.1 ± 2.8	24.4 ± 2.6	0.672 ^†^
Time since the op. (mo)	32 (28–46)	34 (26–48)	36 (30–52)	30 (28–34)	0.101 ^‡^
BCC (N,%)	54 (58.1)	20 (64.5)	18 (60.0)	15 (50.0)	0.502 *
SCC (N,%)	39 (41.9)	11 (35.5)	12 (40.0)	15 (50.0)	
Defect diameter (mm)	32 (22–44)	25 (17–32)	29 (19–33)	49 (42–54)	<0.001 ^‡^

All data are presented as whole numbers (percentages), mean ± standard deviation or median (interquartile range). **Abbreviations: FTSG—**full-thickness skin graft; **STSG—**split-thickness skin graft; **BMI—**body mass index; **BCC—**basal cell carcinoma; **SCC—**squamous cell carcinoma; * chi-square; ^†^ one-way analysis of variance (ANOVA) with post hoc Tukey’s test; ^‡^ Kruskal–Wallis test with post hoc Dunn’s test.

**Table 2:** Comparison of objective skin parameters between the reconstructed site and the healthy contralateral side.

Parameter	Reconstruction Site	Healthy Contralateral Side	p *
**Random Skin Flap (N = 31)**			
Melanin (AU)	113.0 ± 36.3	115.0 ± 37.2	0.836 *
Erythema (AU)	329 (297–343)	322 (283–362)	0.341 ^†^
Hydration (AU)	49.1 ± 14.4	51.1 ± 14.5	0.587 *
Sebum (AU)	28.0 (21.2–56.0)	29.0 (18.0–54.0)	0.760 ^†^
Friction (AU)	138.0 (68.0–189.0)	152.0 (77.0–200.0)	0.371 ^†^
TEWL (g/m^2^/h)	11.5 (10.4–13.3)	11.0 (9.9–13.2)	0.799 ^†^
**FTSG (N = 30)**			
Melanin (AU)	82.4 ± 31.1	74.6 ± 30.3	0.319 *
Erythema (AU)	379 (288–404)	244 (187–291)	<0.001 ^†^
Hydration (AU)	29.0 ± 13.0	36.5 ± 13.7	0.032 *
Sebum (AU)	17.0 (11.0–28.0)	28.0 (24.2–35.0)	<0.001 ^†^
Friction (AU)	82.0 (75.0–129.0)	122.0 (91.0–185.7)	<0.035 ^†^
TEWL (g/m^2^/h)	13.0 (12.0–15.3)	10.9 (9.8–13.0)	<0.001 ^†^
**STSG (N = 30)**			
Melanin (AU)	119.0 ± 28.9	83.1 ± 27.6	<0.001 *
Erythema (AU)	338 (314–396)	222 (181–289)	<0.001 ^†^
Hydration (AU)	19.9 ± 6.3	37.9 ± 8.2	<0.001 *
Sebum (AU)	21.0 (10.0–24.0)	39.0 (41.0–72.0)	<0.001 ^†^
Friction (AU)	81.0 (58.0–137.7)	121.1 (106.0–212.7)	<0.001 ^†^
TEWL (g/m^2^/h)	13.1 (11.8–14.2)	10.8 (9.4–12.3)	<0.001 ^†^

All data are presented as median (IQR). **Abbreviations: FTSG—**full-thickness skin graft; **STSG—**split-thickness skin graft; **TEWL—**transepidermal water loss. * Student’s *t*-test; ^†^ Mann–Whitney U test.

## Data Availability

All data sets are available upon request to the corresponding author.
